# Supplementary material for: Variability of visual field maps in human early extrastriate cortex challenges the canonical model of organization of V2 and V3
Source: eLife. 2023 Aug 15;12:e86439. doi: 10.7554/eLife.86439 (PMC10427147; doi:10.7554/eLife.86439)
Supplement: Supplementary file 1. — This file was exported from Jamovi. [file elife-86439-supp1.pdf]

Results - Polar angle

Mixed Model

Model Info

Info	
Estimate	Linear mixed model fit by REML
Call	Mean difference from the average map ~ 1 + Hemisphere + Visual area + Half + Hemisphere:Visual area + Hemisphere:Half + Visual area:Half + Hemisphere:Visual area:Half+(1 + Hemisphere + Visual area + Half | HCP_ID)
AIC	13759.810
BIC	13917.583
LogLikel.	-6851.224
R-squared Marginal	0.203
R-squared Conditional	0.578
Converged	yes
Optimizer	bobyqa

Note. (Almost) singular fit. Maybe random coefficients variances are too small or correlations among them too large.

Note. boundary (singular) fit: see help('isSingular')

[3]

Model Results

Fixed Effect Omnibus tests

	F	Num df	Den df	p
Hemisphere	109.57	1	181	< .001
Visual area	78.90	2	258	< .001
Half	132.33	1	181	< .001
Hemisphere ✻ Visual area	20.68	2	1440	< .001
Hemisphere ✻ Half	176.74	1	1440	< .001
Visual area ✻ Half	18.66	2	1440	< .001
Hemisphere ✻ Visual area ✻ Half	6.65	2	1440	0.001

Note. Satterthwaite method for degrees of freedom

Fixed Effects Parameter Estimates

Names	Effect	Estimate	SE	95% Confidence Interval		df	t	p
				Lower	Upper			
(Intercept)	(Intercept)	18.584	0.300	17.995	19.173	180	61.859	< .001
Hemisphere1	RH - LH	-3.347	0.320	-3.974	-2.720	181	-10.468	< .001
Visual area1	V2 - V1	2.381	0.290	1.812	2.950	210	8.206	< .001
Visual area2	V3 - V1	3.985	0.318	3.363	4.608	187	12.541	< .001
Half1	ventral - dorsal	-3.301	0.287	-3.864	-2.739	181	-11.504	< .001
Hemisphere1 ✻ Visual area1	RH - LH ✻ V2 - V1	-1.273	0.503	-2.260	-0.287	1440	-2.529	0.012
Hemisphere1 ✻ Visual area2	RH - LH ✻ V3 - V1	1.942	0.503	0.955	2.929	1440	3.857	< .001
Hemisphere1 ✻ Half1	RH - LH ✻ ventral - dorsal	5.465	0.411	4.659	6.271	1440	13.295	< .001
Visual area1 ✻ Half1	V2 - V1 ✻ ventral - dorsal	2.799	0.503	1.812	3.786	1440	5.559	< .001
Visual area2 ✻ Half1	V3 - V1 ✻ ventral - dorsal	2.504	0.503	1.518	3.491	1440	4.974	< .001
Hemisphere1 ✻ Visual area1 ✻ Half1	RH - LH ✻ V2 - V1 ✻ ventral - dorsal	2.561	1.007	0.587	4.534	1440	2.543	0.011
Hemisphere1 ✻ Visual area2 ✻ Half1	RH - LH ✻ V3 - V1 ✻ ventral - dorsal	-0.998	1.007	-2.971	0.976	1440	-0.991	0.322

Random Components

Groups	Name	SD	Variance	ICC
HCP_ID	(Intercept)	3.80	14.42	0.386
	Hemisphere1	3.30	10.86	
	Visual area1	1.94	3.77	
	Visual area2	2.61	6.81	
	Half1	2.69	7.26	
Residual		4.79	22.94	

Note. Number of Obs: 2172 , groups: HCP_ID 181

Random Parameters correlations

Groups	Param.1	Param.2	Corr.
HCP_ID	(Intercept)	Hemisphere1	0.1306
	(Intercept)	Visual area1	-0.3568
	(Intercept)	Visual area2	-0.1894
	(Intercept)	Half1	-0.1298
	Hemisphere1	Visual area1	-0.0893
	Hemisphere1	Visual area2	0.2724
	Hemisphere1	Half1	-0.0742
	Visual area1	Visual area2	0.8750
	Visual area1	Half1	0.2956
	Visual area2	Half1	0.5439

Post Hoc Tests

Post Hoc Comparisons - Hemisphere

Comparison							
Hemisphere		Hemisphere	Difference	SE	t	df	$p_{bonferroni}$
LH	-	RH	3.35	0.320	10.5	180	< .001

Post Hoc Comparisons - Visual area

Comparison							
Visual area		Visual area	Difference	SE	t	df	$p_{bonferroni}$
V1	-	V2	-2.38	0.290	-8.21	180	< .001
V1	-	V3	-3.99	0.318	-12.54	180	< .001
V2	-	V3	-1.60	0.270	-5.95	180	< .001

Post Hoc Comparisons - Half

Comparison							
Half		Half	Difference	SE	t	df	$p_{bonferroni}$
dorsal	-	ventral	3.30	0.287	11.5	180	< .001

Post Hoc Comparisons - Hemisphere ✻ Visual area

Comparison										
Hemisphere	Visual area		Hemisphere	Visual area	Difference	SE	t	df	$p_{bonferroni}$
LH	V1	-	LH	V2	-3.01752	0.384	-7.85547	512	< .001
LH	V1	-	LH	V3	-3.01448	0.405	-7.43566	451	< .001
LH	V1	-	RH	V1	3.56989	0.432	8.26137	547	< .001
LH	V1	-	RH	V2	1.82575	0.449	4.06999	224	< .001
LH	V1	-	RH	V3	-1.38641	0.500	-2.77163	214	0.091
LH	V2	-	LH	V3	0.00305	0.369	0.00825	568	1.000
LH	V2	-	RH	V2	4.84328	0.432	11.20822	547	< .001
LH	V2	-	RH	V3	1.63111	0.478	3.41319	218	0.011
LH	V3	-	RH	V3	1.62807	0.432	3.76764	547	0.003
RH	V1	-	LH	V2	-6.58741	0.462	-14.24498	221	< .001
RH	V1	-	LH	V3	-6.58436	0.445	-14.77990	225	< .001
RH	V1	-	RH	V2	-1.74413	0.384	-4.54048	512	< .001
RH	V1	-	RH	V3	-4.95630	0.405	-12.22545	451	< .001
RH	V2	-	LH	V3	-4.84023	0.405	-11.95180	236	< .001
RH	V2	-	RH	V3	-3.21216	0.369	-8.70360	568	< .001

Post Hoc Comparisons - Hemisphere ✻ Half

Comparison									
Hemisphere	Half		Hemisphere	Half	Difference	SE	t	df	$p_{bonferroni}$
LH	dorsal	-	LH	ventral	6.0338	0.353	17.094	397	< .001
LH	dorsal	-	RH	dorsal	6.0796	0.380	15.995	351	< .001
LH	dorsal	-	RH	ventral	6.6483	0.421	15.789	180	< .001
LH	ventral	-	RH	ventral	0.6145	0.380	1.617	351	0.641
RH	dorsal	-	LH	ventral	-0.0459	0.438	-0.105	180	1.000
RH	dorsal	-	RH	ventral	0.5686	0.353	1.611	397	0.648

Post Hoc Comparisons - Visual area ✻ Half

Comparison									
Visual area	Half		Visual area	Half	Difference	SE	t	df	$p_{bonferroni}$
V1	dorsal	-	V1	ventral	5.069	0.408	12.410	642	< .001
V1	dorsal	-	V2	dorsal	-0.981	0.384	-2.555	512	0.164
V1	dorsal	-	V2	ventral	1.289	0.452	2.848	223	0.072
V1	dorsal	-	V3	dorsal	-2.733	0.405	-6.742	451	< .001
V1	dorsal	-	V3	ventral	-0.169	0.497	-0.340	215	1.000
V1	ventral	-	V2	ventral	-3.780	0.384	-9.841	512	< .001
V1	ventral	-	V3	ventral	-5.238	0.405	-12.919	451	< .001
V2	dorsal	-	V1	ventral	6.050	0.413	14.649	234	< .001
V2	dorsal	-	V2	ventral	2.270	0.408	5.558	642	< .001
V2	dorsal	-	V3	dorsal	-1.752	0.369	-4.747	568	< .001
V2	dorsal	-	V3	ventral	0.813	0.449	1.811	224	1.000
V2	ventral	-	V3	ventral	-1.457	0.369	-3.949	568	0.001
V3	dorsal	-	V1	ventral	7.802	0.403	19.372	237	< .001
V3	dorsal	-	V2	ventral	4.022	0.389	10.345	242	< .001
V3	dorsal	-	V3	ventral	2.565	0.408	6.279	642	< .001

Post Hoc Comparisons - Hemisphere ✻ Visual area ✻ Half

Comparison											
Hemisphere	Visual area	Half		Hemisphere	Visual area	Half	Difference	SE	t	df	$p_{bonferroni}$
LH	V1	dorsal	-	LH	V1	ventral	7.541	0.542	13.918	1179	< .001
LH	V1	dorsal	-	LH	V2	dorsal	-2.258	0.524	-4.312	1106	0.001
LH	V1	dorsal	-	LH	V2	ventral	3.764	0.576	6.538	537	< .001
LH	V1	dorsal	-	LH	V3	dorsal	-1.513	0.540	-2.804	993	0.340
LH	V1	dorsal	-	LH	V3	ventral	3.025	0.611	4.950	465	< .001
LH	V1	dorsal	-	RH	V1	dorsal	6.042	0.560	10.791	1051	< .001
LH	V1	dorsal	-	RH	V1	ventral	8.639	0.588	14.680	608	< .001
LH	V1	dorsal	-	RH	V2	dorsal	6.337	0.573	11.066	544	< .001
LH	V1	dorsal	-	RH	V2	ventral	4.855	0.615	7.898	339	< .001
LH	V1	dorsal	-	RH	V3	dorsal	2.088	0.614	3.401	460	0.048
LH	V1	dorsal	-	RH	V3	ventral	2.680	0.672	3.986	302	0.006
LH	V1	ventral	-	LH	V2	ventral	-3.777	0.524	-7.211	1106	< .001
LH	V1	ventral	-	LH	V3	ventral	-4.516	0.540	-8.370	993	< .001
LH	V1	ventral	-	RH	V1	ventral	1.098	0.560	1.961	1051	1.000
LH	V1	ventral	-	RH	V2	ventral	-2.686	0.573	-4.690	544	< .001
LH	V1	ventral	-	RH	V3	ventral	-4.861	0.614	-7.918	460	< .001
LH	V2	dorsal	-	LH	V1	ventral	9.799	0.545	17.971	625	< .001
LH	V2	dorsal	-	LH	V2	ventral	6.023	0.542	11.115	1179	< .001
LH	V2	dorsal	-	LH	V3	dorsal	0.746	0.513	1.454	1189	1.000
LH	V2	dorsal	-	LH	V3	ventral	5.283	0.573	9.222	544	< .001
LH	V2	dorsal	-	RH	V1	ventral	10.897	0.597	18.255	355	< .001
LH	V2	dorsal	-	RH	V2	dorsal	8.596	0.560	15.353	1051	< .001
LH	V2	dorsal	-	RH	V2	ventral	7.113	0.588	12.088	608	< .001
LH	V2	dorsal	-	RH	V3	dorsal	4.347	0.596	7.294	493	< .001
LH	V2	dorsal	-	RH	V3	ventral	4.938	0.643	7.683	320	< .001
LH	V2	ventral	-	LH	V3	ventral	-0.739	0.513	-1.442	1189	1.000
LH	V2	ventral	-	RH	V2	ventral	1.091	0.560	1.948	1051	1.000
LH	V2	ventral	-	RH	V3	ventral	-1.085	0.596	-1.820	493	1.000
LH	V3	dorsal	-	LH	V1	ventral	9.054	0.538	16.843	653	< .001
LH	V3	dorsal	-	LH	V2	ventral	5.277	0.527	10.011	694	< .001
LH	V3	dorsal	-	LH	V3	ventral	4.538	0.542	8.375	1179	< .001
LH	V3	dorsal	-	RH	V1	ventral	10.152	0.562	18.065	392	< .001
LH	V3	dorsal	-	RH	V2	ventral	6.368	0.546	11.657	414	< .001
LH	V3	dorsal	-	RH	V3	dorsal	3.601	0.560	6.432	1051	< .001
LH	V3	dorsal	-	RH	V3	ventral	4.193	0.588	7.125	608	< .001
LH	V3	ventral	-	RH	V3	ventral	-0.345	0.560	-0.616	1051	1.000
RH	V1	dorsal	-	LH	V1	ventral	1.499	0.601	2.495	573	0.849
RH	V1	dorsal	-	LH	V2	dorsal	-8.300	0.584	-14.223	519	< .001
RH	V1	dorsal	-	LH	V2	ventral	-2.278	0.636	-3.579	324	0.026
RH	V1	dorsal	-	LH	V3	dorsal	-7.555	0.570	-13.248	551	< .001
RH	V1	dorsal	-	LH	V3	ventral	-3.017	0.644	-4.684	319	< .001
RH	V1	dorsal	-	RH	V1	ventral	2.597	0.542	4.793	1179	< .001
RH	V1	dorsal	-	RH	V2	dorsal	0.295	0.524	0.564	1106	1.000
RH	V1	dorsal	-	RH	V2	ventral	-1.187	0.576	-2.062	537	1.000
RH	V1	dorsal	-	RH	V3	dorsal	-3.954	0.540	-7.328	993	< .001
RH	V1	dorsal	-	RH	V3	ventral	-3.362	0.611	-5.502	465	< .001
RH	V1	ventral	-	LH	V2	ventral	-4.875	0.584	-8.352	519	< .001
RH	V1	ventral	-	LH	V3	ventral	-5.614	0.570	-9.844	551	< .001
RH	V1	ventral	-	RH	V2	ventral	-3.784	0.524	-7.225	1106	< .001
RH	V1	ventral	-	RH	V3	ventral	-5.959	0.540	-11.045	993	< .001
RH	V2	dorsal	-	LH	V1	ventral	1.204	0.599	2.011	353	1.000
RH	V2	dorsal	-	LH	V2	ventral	-2.573	0.601	-4.284	573	0.001
RH	V2	dorsal	-	LH	V3	dorsal	-7.850	0.539	-14.559	646	< .001

Post Hoc Comparisons - Hemisphere ✻ Visual area ✻ Half

Comparison											
Hemisphere	Visual area	Half		Hemisphere	Visual area	Half	Difference	SE	t	df	$p_{bonferroni}$
RH	V2	dorsal	-	LH	V3	ventral	-3.313	0.603	-5.496	349	< .001
RH	V2	dorsal	-	RH	V1	ventral	2.301	0.545	4.221	625	0.002
RH	V2	dorsal	-	RH	V2	ventral	-1.482	0.542	-2.736	1179	0.417
RH	V2	dorsal	-	RH	V3	dorsal	-4.249	0.513	-8.286	1189	< .001
RH	V2	dorsal	-	RH	V3	ventral	-3.658	0.573	-6.384	544	< .001
RH	V2	ventral	-	LH	V3	ventral	-1.830	0.539	-3.394	646	0.048
RH	V2	ventral	-	RH	V3	ventral	-2.175	0.513	-4.242	1189	0.002
RH	V3	dorsal	-	LH	V1	ventral	5.453	0.618	8.821	337	< .001
RH	V3	dorsal	-	LH	V2	ventral	1.676	0.614	2.728	340	0.442
RH	V3	dorsal	-	LH	V3	ventral	0.936	0.601	1.559	573	1.000
RH	V3	dorsal	-	RH	V1	ventral	6.550	0.538	12.186	653	< .001
RH	V3	dorsal	-	RH	V2	ventral	2.767	0.527	5.248	694	< .001
RH	V3	dorsal	-	RH	V3	ventral	0.591	0.542	1.091	1179	1.000

References

[1] The jamovi project (2022). *jamovi*. (Version 2.3) [Computer Software]. Retrieved from https://www.jamovi.org.

[2] R Core Team (2021). *R: A Language and environment for statistical computing*. (Version 4.1) [Computer software]. Retrieved from https://cran.r-project.org. (R packages retrieved from MRAN snapshot 2022-01-01).

[3] Gallucci, M. (2019). *GAMLj: General analyses for linear models*. [jamovi module]. Retrieved from https://gamlj.github.io/.
