# Supplementary material for: Variability of visual field maps in human early extrastriate cortex challenges the canonical model of organization of V2 and V3
Source: eLife. 2023 Aug 15;12:e86439. doi: 10.7554/eLife.86439 (PMC10427147; doi:10.7554/eLife.86439)
Supplement: Supplementary file 2. — This file was exported from Jamovi. [file elife-86439-supp2.pdf]

Results - Eccentricity

Mixed Model

Model Info

Info	
Estimate	Linear mixed model fit by REML
Call	Mean difference from the average map ~ 1 + Hemisphere + Visual area + Half + Hemisphere:Visual area + Hemisphere:Half + Visual area:Half + Hemisphere:Visual area:Half+(1 + Hemisphere + Visual area + Half | HCP_ID)
AIC	833.430
BIC	1062.619
LogLikel.	-423.742
R-squared Marginal	0.105
R-squared Conditional	0.671
Converged	yes
Optimizer	bobyqa

Note. (Almost) singular fit. Maybe random coefficients variances are too small or correlations among them too large.

Note. boundary (singular) fit: see help('isSingular')

[3]

Model Results

Fixed Effect Omnibus tests

	F	Num df	Den df	p
Hemisphere	118.93	1	181	< .001
Visual area	5.54	2	259	0.004
Half	21.55	1	180	< .001
Hemisphere ✻ Visual area	16.88	2	1440	< .001
Hemisphere ✻ Half	245.31	1	1440	< .001
Visual area ✻ Half	21.86	2	1440	< .001
Hemisphere ✻ Visual area ✻ Half	8.77	2	1440	< .001

Note. Satterthwaite method for degrees of freedom

Fixed Effects Parameter Estimates

Names	Effect	Estimate	SE	95% Confidence Interval Lower	Upper	df	t	p
(Intercept)	(Intercept)	0.8097	0.0193	0.7718	0.84762	180	41.860	< .001
Hemisphere1	RH - LH	-0.1367	0.0125	-0.1612	-0.11210	181	-10.906	< .001
Visual area1	V2 - V1	0.0133	0.0135	-0.0132	0.03979	402	0.984	0.326
Visual area2	V3 - V1	0.0484	0.0149	0.0191	0.07763	182	3.237	0.001
Half1	ventral - dorsal	-0.1275	0.0275	-0.1814	-0.07369	180	-4.642	< .001
Hemisphere1 ✻ Visual area1	RH - LH ✻ V2 - V1	-0.0136	0.0250	-0.0626	0.03551	1440	-0.542	0.588
Hemisphere1 ✻ Visual area2	RH - LH ✻ V3 - V1	0.1186	0.0250	0.0696	0.16772	1440	4.739	< .001
Hemisphere1 ✻ Half1	RH - LH ✻ ventral - dorsal	0.3202	0.0204	0.2801	0.36026	1440	15.662	< .001
Visual area1 ✻ Half1	V2 - V1 ✻ ventral - dorsal	0.0798	0.0250	0.0307	0.12882	1440	3.185	0.001
Visual area2 ✻ Half1	V3 - V1 ✻ ventral - dorsal	0.1655	0.0250	0.1164	0.21460	1440	6.611	< .001
Hemisphere1 ✻ Visual area1 ✻ Half1	RH - LH ✻ V2 - V1 ✻ ventral - dorsal	0.1161	0.0501	0.0179	0.21423	1440	2.318	0.021
Hemisphere1 ✻ Visual area2 ✻ Half1	RH - LH ✻ V3 - V1 ✻ ventral - dorsal	-0.0933	0.0501	-0.1914	0.00487	1440	-1.863	0.063

Random Components

Groups	Name	SD	Variance	ICC
HCP_ID	(Intercept)	0.2510	0.06299	0.526
	Hemisphere1	0.0975	0.00951	
	Visual area1	0.0685	0.00470	
	Visual area2	0.1096	0.01202	
	Half1	0.3431	0.11771	
Residual		0.2382	0.05673	

Note. Number of Obs: 2172 , groups: HCP_ID 181

Random Parameters correlations

Groups	Param.1	Param.2	Corr.
HCP_ID	(Intercept)	Hemisphere1	-0.33880
	(Intercept)	Visual area1	0.76019
	(Intercept)	Visual area2	0.12968
	(Intercept)	Half1	-0.76439
	Hemisphere1	Visual area1	-0.20279
	Hemisphere1	Visual area2	0.17127
	Hemisphere1	Half1	0.19810
	Visual area1	Visual area2	0.59015
	Visual area1	Half1	-0.78789
	Visual area2	Half1	0.00116

Post Hoc Tests

Post Hoc Comparisons - Hemisphere

Comparison							
Hemisphere		Hemisphere	Difference	SE	t	df	$p_{bonferroni}$
LH	-	RH	0.137	0.0125	10.9	180	< .001

Post Hoc Comparisons - Visual area

Comparison							
Visual area		Visual area	Difference	SE	t	df	$p_{bonferroni}$
V1	-	V2	-0.0133	0.0135	-0.984	180	0.980
V1	-	V3	-0.0484	0.0149	-3.237	180	0.004
V2	-	V3	-0.0351	0.0141	-2.478	180	0.042

Post Hoc Comparisons - Half

Comparison							
Half		Half	Difference	SE	t	df	$p_{bonferroni}$
dorsal	-	ventral	0.128	0.0275	4.64	180	< .001

Post Hoc Comparisons - Hemisphere ✻ Visual area

Comparison										
Hemisphere	Visual area		Hemisphere	Visual area	Difference	SE	t	df	$p_{bonferroni}$
LH	V1	-	LH	V2	-0.02008	0.0184	-1.090	562	1.000
LH	V1	-	LH	V3	0.01097	0.0195	0.563	487	1.000
LH	V1	-	RH	V1	0.17169	0.0191	8.974	780	< .001
LH	V1	-	RH	V2	0.16518	0.0194	8.507	242	< .001
LH	V1	-	RH	V3	0.06402	0.0213	3.009	229	0.044
LH	V2	-	LH	V3	0.03105	0.0189	1.644	526	1.000
LH	V2	-	RH	V2	0.18525	0.0191	9.683	780	< .001
LH	V2	-	RH	V3	0.08410	0.0211	3.988	231	0.001
LH	V3	-	RH	V3	0.05305	0.0191	2.773	780	0.085
RH	V1	-	LH	V2	-0.19177	0.0202	-9.506	236	< .001
RH	V1	-	LH	V3	-0.16072	0.0203	-7.916	235	< .001
RH	V1	-	RH	V2	-0.00652	0.0184	-0.354	562	1.000
RH	V1	-	RH	V3	-0.10767	0.0195	-5.525	487	< .001
RH	V2	-	LH	V3	-0.15421	0.0193	-7.972	242	< .001
RH	V2	-	RH	V3	-0.10116	0.0189	-5.355	526	< .001

Post Hoc Comparisons - Hemisphere ✻ Half

Comparison									
Hemisphere	Half		Hemisphere	Half	Difference	SE	t	df	$p_{bonferroni}$
LH	dorsal	-	LH	ventral	0.28763	0.0293	9.812	233	< .001
LH	dorsal	-	RH	dorsal	0.29676	0.0162	18.351	470	< .001
LH	dorsal	-	RH	ventral	0.26420	0.0314	8.418	180	< .001
LH	ventral	-	RH	ventral	-0.02343	0.0162	-1.449	470	0.888
RH	dorsal	-	LH	ventral	-0.00913	0.0290	-0.315	180	1.000
RH	dorsal	-	RH	ventral	-0.03256	0.0293	-1.111	233	1.000

Post Hoc Comparisons - Visual area ✻ Half

Comparison									
Visual area	Half		Visual area	Half	Difference	SE	t	df	$p_{bonferroni}$
V1	dorsal	-	V1	ventral	0.20930	0.0310	6.742	290	< .001
V1	dorsal	-	V2	dorsal	0.02658	0.0184	1.443	562	1.000
V1	dorsal	-	V2	ventral	0.15612	0.0280	5.572	206	< .001
V1	dorsal	-	V3	dorsal	0.03441	0.0195	1.765	487	1.000
V1	dorsal	-	V3	ventral	0.07818	0.0321	2.435	200	0.236
V1	ventral	-	V2	ventral	-0.05317	0.0184	-2.886	562	0.061
V1	ventral	-	V3	ventral	-0.13111	0.0195	-6.727	487	< .001
V2	dorsal	-	V1	ventral	0.18272	0.0346	5.287	197	< .001
V2	dorsal	-	V2	ventral	0.12955	0.0310	4.173	290	< .001
V2	dorsal	-	V3	dorsal	0.00783	0.0189	0.415	526	1.000
V2	dorsal	-	V3	ventral	0.05160	0.0348	1.482	197	1.000
V2	ventral	-	V3	ventral	-0.07794	0.0189	-4.126	526	< .001
V3	dorsal	-	V1	ventral	0.17489	0.0321	5.450	200	< .001
V3	dorsal	-	V2	ventral	0.12171	0.0283	4.298	206	< .001
V3	dorsal	-	V3	ventral	0.04377	0.0310	1.410	290	1.000

Post Hoc Comparisons - Hemisphere ✻ Visual area ✻ Half

Comparison											
Hemisphere	Visual area	Half		Hemisphere	Visual area	Half	Difference	SE	t	df	$p_{bonferroni}$
LH	V1	dorsal	-	LH	V1	ventral	0.36559	0.0357	10.230	482	< .001
LH	V1	dorsal	-	LH	V2	dorsal	-0.00922	0.0256	-0.361	1181	1.000
LH	V1	dorsal	-	LH	V2	ventral	0.33466	0.0331	10.097	391	< .001
LH	V1	dorsal	-	LH	V3	dorsal	0.11705	0.0263	4.445	1063	< .001
LH	V1	dorsal	-	LH	V3	ventral	0.27048	0.0367	7.378	333	< .001
LH	V1	dorsal	-	RH	V1	dorsal	0.32799	0.0261	12.583	1306	< .001
LH	V1	dorsal	-	RH	V1	ventral	0.38099	0.0375	10.171	356	< .001
LH	V1	dorsal	-	RH	V2	dorsal	0.39037	0.0263	14.856	689	< .001
LH	V1	dorsal	-	RH	V2	ventral	0.30558	0.0348	8.787	290	< .001
LH	V1	dorsal	-	RH	V3	dorsal	0.27975	0.0277	10.107	589	< .001
LH	V1	dorsal	-	RH	V3	ventral	0.21387	0.0386	5.540	263	< .001
LH	V1	ventral	-	LH	V2	ventral	-0.03093	0.0256	-1.211	1181	1.000
LH	V1	ventral	-	LH	V3	ventral	-0.09511	0.0263	-3.612	1063	0.021
LH	V1	ventral	-	RH	V1	ventral	0.01540	0.0261	0.591	1306	1.000
LH	V1	ventral	-	RH	V2	ventral	-0.06001	0.0263	-2.284	689	1.000
LH	V1	ventral	-	RH	V3	ventral	-0.15172	0.0277	-5.481	589	< .001
LH	V2	dorsal	-	LH	V1	ventral	0.37481	0.0388	9.652	309	< .001
LH	V2	dorsal	-	LH	V2	ventral	0.34388	0.0357	9.622	482	< .001
LH	V2	dorsal	-	LH	V3	dorsal	0.12627	0.0259	4.877	1129	< .001
LH	V2	dorsal	-	LH	V3	ventral	0.27970	0.0391	7.161	307	< .001
LH	V2	dorsal	-	RH	V1	ventral	0.39021	0.0406	9.610	253	< .001
LH	V2	dorsal	-	RH	V2	dorsal	0.39959	0.0261	15.330	1306	< .001
LH	V2	dorsal	-	RH	V2	ventral	0.31480	0.0375	8.404	356	< .001
LH	V2	dorsal	-	RH	V3	dorsal	0.28898	0.0275	10.496	598	< .001
LH	V2	dorsal	-	RH	V3	ventral	0.22310	0.0411	5.432	251	< .001
LH	V2	ventral	-	LH	V3	ventral	-0.06418	0.0259	-2.479	1129	0.879
LH	V2	ventral	-	RH	V2	ventral	-0.02908	0.0261	-1.116	1306	1.000
LH	V2	ventral	-	RH	V3	ventral	-0.12079	0.0275	-4.387	598	< .001
LH	V3	dorsal	-	LH	V1	ventral	0.24854	0.0366	6.782	333	< .001
LH	V3	dorsal	-	LH	V2	ventral	0.21761	0.0334	6.516	385	< .001
LH	V3	dorsal	-	LH	V3	ventral	0.15343	0.0357	4.293	482	0.001
LH	V3	dorsal	-	RH	V1	ventral	0.26394	0.0381	6.934	266	< .001
LH	V3	dorsal	-	RH	V2	ventral	0.18853	0.0347	5.429	290	< .001
LH	V3	dorsal	-	RH	V3	dorsal	0.16270	0.0261	6.242	1306	< .001
LH	V3	dorsal	-	RH	V3	ventral	0.09682	0.0375	2.585	356	0.669
LH	V3	ventral	-	RH	V3	ventral	-0.05661	0.0261	-2.172	1306	1.000
RH	V1	dorsal	-	LH	V1	ventral	0.03760	0.0354	1.061	390	1.000
RH	V1	dorsal	-	LH	V2	dorsal	-0.33721	0.0268	-12.564	645	< .001
RH	V1	dorsal	-	LH	V2	ventral	0.00667	0.0331	0.202	307	1.000
RH	V1	dorsal	-	LH	V3	dorsal	-0.21093	0.0269	-7.831	638	< .001
RH	V1	dorsal	-	LH	V3	ventral	-0.05751	0.0361	-1.593	279	1.000
RH	V1	dorsal	-	RH	V1	ventral	0.05300	0.0357	1.483	482	1.000
RH	V1	dorsal	-	RH	V2	dorsal	0.06238	0.0256	2.441	1181	0.975
RH	V1	dorsal	-	RH	V2	ventral	-0.02241	0.0331	-0.676	391	1.000
RH	V1	dorsal	-	RH	V3	dorsal	-0.04823	0.0263	-1.832	1063	1.000
RH	V1	dorsal	-	RH	V3	ventral	-0.11411	0.0367	-3.113	333	0.133
RH	V1	ventral	-	LH	V2	ventral	-0.04633	0.0268	-1.726	645	1.000
RH	V1	ventral	-	LH	V3	ventral	-0.11051	0.0269	-4.102	638	0.003
RH	V1	ventral	-	RH	V2	ventral	-0.07541	0.0256	-2.951	1181	0.213
RH	V1	ventral	-	RH	V3	ventral	-0.16712	0.0263	-6.347	1063	< .001
RH	V2	dorsal	-	LH	V1	ventral	-0.02478	0.0384	-0.646	264	1.000
RH	V2	dorsal	-	LH	V2	ventral	-0.05571	0.0354	-1.572	390	1.000
RH	V2	dorsal	-	LH	V3	dorsal	-0.27331	0.0262	-10.423	693	< .001

Post Hoc Comparisons - Hemisphere ✻ Visual area ✻ Half

Comparison											
Hemisphere	Visual area	Half		Hemisphere	Visual area	Half	Difference	SE	t	df	$p_{bonferroni}$
RH	V2	dorsal	-	LH	V3	ventral	-0.11989	0.0383	-3.127	264	0.130
RH	V2	dorsal	-	RH	V1	ventral	-0.00938	0.0388	-0.241	309	1.000
RH	V2	dorsal	-	RH	V2	ventral	-0.08479	0.0357	-2.372	482	1.000
RH	V2	dorsal	-	RH	V3	dorsal	-0.11061	0.0259	-4.272	1129	0.001
RH	V2	dorsal	-	RH	V3	ventral	-0.17649	0.0391	-4.518	307	< .001
RH	V2	ventral	-	LH	V3	ventral	-0.03510	0.0262	-1.338	693	1.000
RH	V2	ventral	-	RH	V3	ventral	-0.09170	0.0259	-3.542	1129	0.027
RH	V3	dorsal	-	LH	V1	ventral	0.08584	0.0366	2.342	275	1.000
RH	V3	dorsal	-	LH	V2	ventral	0.05490	0.0336	1.633	301	1.000
RH	V3	dorsal	-	LH	V3	ventral	-0.00927	0.0354	-0.262	390	1.000
RH	V3	dorsal	-	RH	V1	ventral	0.10123	0.0366	2.762	333	0.400
RH	V3	dorsal	-	RH	V2	ventral	0.02582	0.0334	0.773	385	1.000
RH	V3	dorsal	-	RH	V3	ventral	-0.06588	0.0357	-1.843	482	1.000

References

[1] The jamovi project (2022). *jamovi*. (Version 2.3) [Computer Software]. Retrieved from https://www.jamovi.org.

[2] R Core Team (2021). *R: A Language and environment for statistical computing*. (Version 4.1) [Computer software]. Retrieved from https://cran.r-project.org. (R packages retrieved from MRAN snapshot 2022-01-01).

[3] Gallucci, M. (2019). *GAMLj: General analyses for linear models*. [jamovi module]. Retrieved from https://gamlj.github.io/.
