# Supplementary material for: Variability of visual field maps in human early extrastriate cortex challenges the canonical model of organization of V2 and V3
Source: eLife. 2023 Aug 15;12:e86439. doi: 10.7554/eLife.86439 (PMC10427147; doi:10.7554/eLife.86439)
Supplement: Supplementary file 3. — This file was exported from Jamovi. [file elife-86439-supp3.pdf]

Results - Polar angle

Mixed Model

Model Info

Info	
Estimate	Linear mixed model fit by REML
Call	Mean difference from the average map ~ 1 + Hemisphere + Visual area + Half + Mean difference in curvature + Mean difference in normalized mean BOLD + Mean intra-individual variability + Hemisphere:Visual area + Hemisphere:Half + Visual area:Half + Hemisphere:Visual area:Half+(Hemisphere + Visual area + Half + 1 | HCP_ID)
AIC	13480.166
BIC	13661.947
LogLikel.	-6711.881
R-squared Marginal	0.342
R-squared Conditional	0.626
Converged	yes
Optimizer	bobyqa

Note. (Almost) singular fit. Maybe random coefficients variances are too small or correlations among them too large.

Note. boundary (singular) fit: see help('isSingular')

[3]

Model Results

Fixed Effect Omnibus tests

	F	Num df	Den df	p
Hemisphere	75.80	1	189	< .001
Visual area	66.33	2	384	< .001
Half	51.82	1	207	< .001
Mean difference in curvature	7.97	1	1812	0.005
Mean difference in normalized mean BOLD	1.23	1	597	0.268
Mean intra-individual variability	317.35	1	1364	< .001
Hemisphere ✻ Visual area	6.47	2	1461	0.002
Hemisphere ✻ Half	87.07	1	1518	< .001
Visual area ✻ Half	10.47	2	1451	< .001
Hemisphere ✻ Visual area ✻ Half	1.12	2	1454	0.327

Note. Satterthwaite method for degrees of freedom

Fixed Effects Parameter Estimates

Names	Effect	Estimate	SE	95% Confidence Interval Lower	Upper	df	t	p
(Intercept)	(Intercept)	18.584	0.267	18.0604	19.107	179	69.581	< .001
Hemisphere1	RH - LH	-2.437	0.280	-2.9855	-1.888	189	-8.707	< .001
Visual area1	V2 - V1	1.929	0.327	1.2881	2.571	323	5.897	< .001
Visual area2	V3 - V1	3.538	0.315	2.9200	4.157	288	11.217	< .001
Half1	ventral - dorsal	-2.115	0.294	-2.6903	-1.539	207	-7.199	< .001
Mean difference in curvature	Mean difference in curvature	0.402	0.142	0.1229	0.681	1812	2.823	0.005
Mean difference in normalized mean BOLD	Mean difference in normalized mean BOLD	0.206	0.186	-0.1588	0.572	597	1.108	0.268
Mean intra-individual variability	Mean intra-individual variability	2.902	0.163	2.5829	3.222	1364	17.814	< .001
Hemisphere1 ✻ Visual area1	RH - LH ✻ V2 - V1	-0.838	0.479	-1.7756	0.100	1442	-1.750	0.080
Hemisphere1 ✻ Visual area2	RH - LH ✻ V3 - V1	0.913	0.483	-0.0328	1.860	1462	1.892	0.059
Hemisphere1 ✻ Half1	RH - LH ✻ ventral - dorsal	3.750	0.402	2.9624	4.538	1518	9.331	< .001
Visual area1 ✻ Half1	V2 - V1 ✻ ventral - dorsal	1.968	0.481	1.0258	2.910	1455	4.094	< .001
Visual area2 ✻ Half1	V3 - V1 ✻ ventral - dorsal	1.843	0.481	0.9005	2.786	1456	3.832	< .001
Hemisphere1 ✻ Visual area1 ✻ Half1	RH - LH ✻ V2 - V1 ✻ ventral - dorsal	1.364	0.958	-0.5142	3.242	1446	1.423	0.155
Hemisphere1 ✻ Visual area2 ✻ Half1	RH - LH ✻ V3 - V1 ✻ ventral - dorsal	0.291	0.959	-1.5896	2.171	1448	0.303	0.762

Random Components

Groups	Name	SD	Variance	ICC
HCP_ID	(Intercept)	3.35	11.19	0.351
	Hemisphere1	2.61	6.83	
	Visual area1	1.93	3.71	
	Visual area2	1.89	3.59	
	Half1	2.72	7.40	
Residual		4.55	20.67	

Note. Number of Obs: 2172 , groups: HCP_ID 181

Random Parameters correlations

Groups	Param.1	Param.2	Corr.
HCP_ID	(Intercept)	Hemisphere1	0.0951
	(Intercept)	Visual area1	-0.1737
	(Intercept)	Visual area2	0.0738
	(Intercept)	Half1	-0.1242
	Hemisphere1	Visual area1	-0.1392
	Hemisphere1	Visual area2	0.2809
	Hemisphere1	Half1	0.0240
	Visual area1	Visual area2	0.8619
	Visual area1	Half1	-0.2336
	Visual area2	Half1	-0.0152

Post Hoc Tests

Post Hoc Comparisons - Hemisphere

Comparison							
Hemisphere		Hemisphere	Difference	SE	t	df	$p_{bonferroni}$
LH	-	RH	2.44	0.280	8.70	190	< .001

Post Hoc Comparisons - Visual area

Comparison							
Visual area		Visual area	Difference	SE	t	df	$p_{bonferroni}$
V1	-	V2	-1.93	0.328	-5.88	304	< .001
V1	-	V3	-3.54	0.316	-11.18	273	< .001
V2	-	V3	-1.61	0.252	-6.39	183	< .001

Post Hoc Comparisons - Half

Comparison							
Half		Half	Difference	SE	t	df	$p_{bonferroni}$
dorsal	-	ventral	2.11	0.294	7.19	211	< .001

Post Hoc Comparisons - Hemisphere ✻ Visual area

Comparison										
Hemisphere	Visual area		Hemisphere	Visual area	Difference	SE	t	df	$p_{bonferroni}$
LH	V1	-	LH	V2	-2.348	0.409	-5.74	654	< .001
LH	V1	-	LH	V3	-3.082	0.390	-7.91	589	< .001
LH	V1	-	RH	V1	2.462	0.395	6.23	653	< .001
LH	V1	-	RH	V2	0.952	0.437	2.18	293	0.454
LH	V1	-	RH	V3	-1.533	0.460	-3.33	272	0.015
LH	V2	-	LH	V3	-0.733	0.352	-2.08	617	0.564
LH	V2	-	RH	V2	3.300	0.400	8.25	676	< .001
LH	V2	-	RH	V3	0.815	0.428	1.90	230	0.872
LH	V3	-	RH	V3	1.549	0.391	3.97	633	0.001
RH	V1	-	LH	V2	-4.810	0.475	-10.12	328	< .001
RH	V1	-	LH	V3	-5.544	0.425	-13.04	294	< .001
RH	V1	-	RH	V2	-1.511	0.404	-3.74	634	0.003
RH	V1	-	RH	V3	-3.995	0.406	-9.84	645	< .001
RH	V2	-	LH	V3	-4.033	0.370	-10.89	252	< .001
RH	V2	-	RH	V3	-2.484	0.349	-7.13	600	< .001

Post Hoc Comparisons - Hemisphere ✻ Half

Comparison									
Hemisphere	Half		Hemisphere	Half	Difference	SE	t	df	$p_{bonferroni}$
LH	dorsal	-	LH	ventral	3.990	0.364	10.950	461	< .001
LH	dorsal	-	RH	dorsal	4.312	0.352	12.259	445	< .001
LH	dorsal	-	RH	ventral	4.551	0.417	10.910	215	< .001
LH	ventral	-	RH	ventral	0.562	0.338	1.664	391	0.582
RH	dorsal	-	LH	ventral	-0.322	0.395	-0.817	186	1.000
RH	dorsal	-	RH	ventral	0.240	0.348	0.688	404	1.000

Post Hoc Comparisons - Visual area ✻ Half

Comparison									
Visual area	Half		Visual area	Half	Difference	SE	t	df	$p_{bonferroni}$
V1	dorsal	-	V1	ventral	3.385	0.410	8.253	682	< .001
V1	dorsal	-	V2	dorsal	-0.946	0.405	-2.336	640	0.297
V1	dorsal	-	V2	ventral	0.472	0.420	1.124	262	1.000
V1	dorsal	-	V3	dorsal	-2.617	0.391	-6.696	591	< .001
V1	dorsal	-	V3	ventral	-1.075	0.432	-2.488	252	0.202
V1	ventral	-	V2	ventral	-2.913	0.409	-7.120	655	< .001
V1	ventral	-	V3	ventral	-4.460	0.404	-11.040	639	< .001
V2	dorsal	-	V1	ventral	4.330	0.504	8.599	363	< .001
V2	dorsal	-	V2	ventral	1.417	0.402	3.528	648	0.007
V2	dorsal	-	V3	dorsal	-1.671	0.348	-4.804	596	< .001
V2	dorsal	-	V3	ventral	-0.129	0.427	-0.303	248	1.000
V2	ventral	-	V3	ventral	-1.546	0.347	-4.460	591	< .001
V3	dorsal	-	V1	ventral	6.002	0.476	12.610	342	< .001
V3	dorsal	-	V2	ventral	3.088	0.389	7.934	246	< .001
V3	dorsal	-	V3	ventral	1.542	0.401	3.850	641	0.002

Post Hoc Comparisons - Hemisphere ✻ Visual area ✻ Half

Comparison											
**Hemisphere**	**Visual area**	**Half**		**Hemisphere**	**Visual area**	**Half**	**Difference**	**SE**	**t**	**df**	**$p_{bonferroni}$**
LH	V1	dorsal	-	LH	V1	ventral	4.98410	0.543	9.1731	1236	< .001
LH	V1	dorsal	-	LH	V2	dorsal	-1.70535	0.528	-3.2314	1176	0.084
LH	V1	dorsal	-	LH	V2	ventral	1.99298	0.547	3.6452	656	0.019
LH	V1	dorsal	-	LH	V3	dorsal	-2.23280	0.512	-4.3641	1140	< .001
LH	V1	dorsal	-	LH	V3	ventral	1.05371	0.555	1.8986	608	1.000
LH	V1	dorsal	-	RH	V1	dorsal	4.06140	0.528	7.6920	1219	< .001
LH	V1	dorsal	-	RH	V1	ventral	5.84705	0.583	10.0378	695	< .001
LH	V1	dorsal	-	RH	V2	dorsal	3.87564	0.567	6.8364	715	< .001
LH	V1	dorsal	-	RH	V2	ventral	3.01149	0.571	5.2716	397	< .001
LH	V1	dorsal	-	RH	V3	dorsal	1.06064	0.571	1.8588	589	1.000
LH	V1	dorsal	-	RH	V3	ventral	0.85779	0.602	1.4246	364	1.000
LH	V1	ventral	-	LH	V2	ventral	-2.99112	0.534	-5.6034	1193	< .001
LH	V1	ventral	-	LH	V3	ventral	-3.93039	0.525	-7.4926	1175	< .001
LH	V1	ventral	-	RH	V1	ventral	0.86295	0.516	1.6709	1165	1.000
LH	V1	ventral	-	RH	V2	ventral	-1.97261	0.553	-3.5646	660	0.026
LH	V1	ventral	-	RH	V3	ventral	-4.12631	0.576	-7.1695	601	< .001
LH	V2	dorsal	-	LH	V1	ventral	6.68945	0.626	10.6892	745	< .001
LH	V2	dorsal	-	LH	V2	ventral	3.69833	0.538	6.8753	1214	< .001
LH	V2	dorsal	-	LH	V3	dorsal	-0.52745	0.493	-1.0707	1253	1.000
LH	V2	dorsal	-	LH	V3	ventral	2.75906	0.562	4.9082	644	< .001
LH	V2	dorsal	-	RH	V1	ventral	7.55240	0.668	11.3011	487	< .001
LH	V2	dorsal	-	RH	V2	dorsal	5.58099	0.543	10.2739	1284	< .001
LH	V2	dorsal	-	RH	V2	ventral	4.71685	0.578	8.1591	683	< .001
LH	V2	dorsal	-	RH	V3	dorsal	2.76599	0.551	5.0230	568	< .001
LH	V2	dorsal	-	RH	V3	ventral	2.56314	0.612	4.1899	369	0.002
LH	V2	ventral	-	LH	V3	ventral	-0.93927	0.486	-1.9331	1225	1.000
LH	V2	ventral	-	RH	V2	ventral	1.01851	0.517	1.9702	1168	1.000
LH	V2	ventral	-	RH	V3	ventral	-1.13519	0.544	-2.0875	547	1.000
LH	V3	dorsal	-	LH	V1	ventral	7.21690	0.578	12.4954	671	< .001
LH	V3	dorsal	-	LH	V2	ventral	4.22578	0.515	8.1977	656	< .001
LH	V3	dorsal	-	LH	V3	ventral	3.28651	0.525	6.2617	1167	< .001
LH	V3	dorsal	-	RH	V1	ventral	8.07985	0.602	13.4198	448	< .001
LH	V3	dorsal	-	RH	V2	ventral	5.24430	0.532	9.8526	408	< .001
LH	V3	dorsal	-	RH	V3	dorsal	3.29344	0.517	6.3654	1171	< .001
LH	V3	dorsal	-	RH	V3	ventral	3.09059	0.560	5.5227	625	< .001
LH	V3	ventral	-	RH	V3	ventral	-0.19592	0.516	-0.3796	1165	1.000
RH	V1	dorsal	-	LH	V1	ventral	0.92271	0.556	1.6595	638	1.000
RH	V1	dorsal	-	LH	V2	dorsal	-5.76675	0.592	-9.7469	693	< .001
RH	V1	dorsal	-	LH	V2	ventral	-2.06841	0.581	-3.5603	386	0.027
RH	V1	dorsal	-	LH	V3	dorsal	-6.29420	0.540	-11.6460	675	< .001
RH	V1	dorsal	-	LH	V3	ventral	-3.00769	0.566	-5.3167	385	< .001
RH	V1	dorsal	-	RH	V1	ventral	1.78566	0.524	3.4070	1164	0.045
RH	V1	dorsal	-	RH	V2	dorsal	-0.18575	0.528	-0.3515	1187	1.000
RH	V1	dorsal	-	RH	V2	ventral	-1.04990	0.536	-1.9577	627	1.000
RH	V1	dorsal	-	RH	V3	dorsal	-3.00076	0.527	-5.6920	1176	< .001
RH	V1	dorsal	-	RH	V3	ventral	-3.20361	0.549	-5.8368	595	< .001
RH	V1	ventral	-	LH	V2	ventral	-3.85407	0.578	-6.6693	649	< .001
RH	V1	ventral	-	LH	V3	ventral	-4.79334	0.547	-8.7655	694	< .001
RH	V1	ventral	-	RH	V2	ventral	-2.83556	0.528	-5.3732	1177	< .001
RH	V1	ventral	-	RH	V3	ventral	-4.98926	0.530	-9.4224	1182	< .001
RH	V2	dorsal	-	LH	V1	ventral	1.10846	0.617	1.7951	432	1.000
RH	V2	dorsal	-	LH	V2	ventral	-1.88266	0.555	-3.3908	635	0.049
RH	V2	dorsal	-	LH	V3	dorsal	-6.10844	0.510	-11.9715	747	< .001

Post Hoc Comparisons - Hemisphere ✻ Visual area ✻ Half

Comparison											
Hemisphere	Visual area	Half		Hemisphere	Visual area	Half	Difference	SE	t	df	$p_{bonferroni}$
RH	V2	dorsal	-	LH	V3	ventral	-2.82193	0.555	-5.0859	388	< .001
RH	V2	dorsal	-	RH	V1	ventral	1.97141	0.597	3.3011	658	0.067
RH	V2	dorsal	-	RH	V2	ventral	-0.86415	0.525	-1.6453	1167	1.000
RH	V2	dorsal	-	RH	V3	dorsal	-2.81500	0.491	-5.7386	1249	< .001
RH	V2	dorsal	-	RH	V3	ventral	-3.01785	0.542	-5.5703	581	< .001
RH	V2	ventral	-	LH	V3	ventral	-1.95779	0.499	-3.9250	700	0.006
RH	V2	ventral	-	RH	V3	ventral	-2.15371	0.484	-4.4523	1212	< .001
RH	V3	dorsal	-	LH	V1	ventral	3.92347	0.629	6.2401	428	< .001
RH	V3	dorsal	-	LH	V2	ventral	0.93235	0.569	1.6383	359	1.000
RH	V3	dorsal	-	LH	V3	ventral	-0.00693	0.559	-0.0124	648	1.000
RH	V3	dorsal	-	RH	V1	ventral	4.78641	0.591	8.1016	709	< .001
RH	V3	dorsal	-	RH	V2	ventral	1.95086	0.518	3.7681	665	0.012
RH	V3	dorsal	-	RH	V3	ventral	-0.20285	0.524	-0.3873	1164	1.000

References

[1] The jamovi project (2022). *jamovi*. (Version 2.3) [Computer Software]. Retrieved from https://www.jamovi.org.

[2] R Core Team (2021). *R: A Language and environment for statistical computing*. (Version 4.1) [Computer software]. Retrieved from https://cran.r-project.org. (R packages retrieved from MRAN snapshot 2022-01-01).

[3] Gallucci, M. (2019). *GAMLj: General analyses for linear models*. [jamovi module]. Retrieved from https://gamlj.github.io/.
