# Supplementary material for: Variability of visual field maps in human early extrastriate cortex challenges the canonical model of organization of V2 and V3
Source: eLife. 2023 Aug 15;12:e86439. doi: 10.7554/eLife.86439 (PMC10427147; doi:10.7554/eLife.86439)
Supplement: Supplementary file 4. — This file was exported from Jamovi. [file elife-86439-supp4.pdf]

Results - Eccentricity

Mixed Model

Model Info

Info	
Estimate	Linear mixed model fit by REML
Call	Mean difference from the average map ~ 1 + Hemisphere + Visual area + Half + Mean difference in curvature + Mean difference in normalized mean BOLD + Mean intra-individual variability + Hemisphere:Visual area + Hemisphere:Half + Visual area:Half + Hemisphere:Visual area:Half+(1 + Hemisphere + Visual area + Half | HCP_ID)
AIC	249.170
BIC	522.536
LogLikel.	-142.175
R-squared Marginal	0.364
R-squared Conditional	0.725
Converged	yes
Optimizer	bobyqa

Note. (Almost) singular fit. Maybe random coefficients variances are too small or correlations among them too large.

Note. boundary (singular) fit: see help('isSingular')

[3]

Model Results

Fixed Effect Omnibus tests

	F	Num df	Den df	p
Hemisphere	40.99	1	201	< .001
Visual area	1.20	2	312	0.303
Half	10.48	1	186	0.001
Mean difference in curvature	2.32	1	1829	0.128
Mean difference in normalized mean BOLD	5.49	1	489	0.020
Mean intra-individual variability	702.87	1	1982	< .001
Hemisphere ✻ Visual area	1.34	2	1463	0.262
Hemisphere ✻ Half	73.49	1	1553	< .001
Visual area ✻ Half	3.29	2	1462	0.038
Hemisphere ✻ Visual area ✻ Half	2.14	2	1447	0.118

Note. Satterthwaite method for degrees of freedom

Fixed Effects Parameter Estimates

Names	Effect	Estimate	SE	95% Confidence Interval		df	t	p
				Lower	Upper			
(Intercept)	(Intercept)	0.80970	0.01482	0.78066	0.83875	179	54.642	< .001
Hemisphere1	RH - LH	-0.06974	0.01089	-0.09110	-0.04839	201	-6.402	< .001
Visual area1	V2 - V1	-0.02229	0.01464	-0.05098	0.00640	506	-1.523	0.128
Visual area2	V3 - V1	-0.01960	0.01633	-0.05160	0.01241	262	-1.200	0.231
Half1	ventral - dorsal	-0.06971	0.02154	-0.11192	-0.02750	186	-3.237	0.001
Mean difference in curvature	Mean difference in curvature	0.01010	0.00662	-0.00289	0.02308	1829	1.524	0.128
Mean difference in normalized mean BOLD	Mean difference in normalized mean BOLD	0.01856	0.00792	0.00304	0.03408	489	2.343	0.020
Mean intra-individual variability	Mean intra-individual variability	0.21122	0.00797	0.19560	0.22683	1982	26.512	< .001
Hemisphere1 ✻ Visual area1	RH - LH ✻ V2 - V1	-0.00710	0.02202	-0.05026	0.03605	1439	-0.323	0.747
Hemisphere1 ✻ Visual area2	RH - LH ✻ V3 - V1	0.02780	0.02236	-0.01603	0.07162	1472	1.243	0.214
Hemisphere1 ✻ Half1	RH - LH ✻ ventral - dorsal	0.16236	0.01894	0.12524	0.19948	1553	8.573	< .001
Visual area1 ✻ Half1	V2 - V1 ✻ ventral - dorsal	0.02676	0.02212	-0.01660	0.07012	1451	1.210	0.227
Visual area2 ✻ Half1	V3 - V1 ✻ ventral - dorsal	0.05757	0.02246	0.01354	0.10160	1485	2.563	0.010
Hemisphere1 ✻ Visual area1 ✻ Half1	RH - LH ✻ V2 - V1 ✻ ventral - dorsal	0.01609	0.04419	-0.07052	0.10270	1447	0.364	0.716
Hemisphere1 ✻ Visual area2 ✻ Half1	RH - LH ✻ V3 - V1 ✻ ventral - dorsal	-0.06999	0.04409	-0.15641	0.01644	1442	-1.587	0.113

Random Components

Groups	Name	SD	Variance	ICC
HCP_ID	(Intercept)	0.1900	0.03609	0.451
	Hemisphere1	0.0755	0.00570	
	Visual area1	0.0719	0.00517	
	Visual area2	0.1289	0.01661	
	Half1	0.2588	0.06695	
Residual		0.2094	0.04384	

Note. Number of Obs: 2172 , groups: HCP_ID 181

Random Parameters correlations

Groups	Param.1	Param.2	Corr.
HCP_ID	(Intercept)	Hemisphere1	-0.1126
	(Intercept)	Visual area1	0.2636
	(Intercept)	Visual area2	-0.1896
	(Intercept)	Half1	-0.6842
	Hemisphere1	Visual area1	-0.2453
	Hemisphere1	Visual area2	0.0909
	Hemisphere1	Half1	0.1587
	Visual area1	Visual area2	0.7024
	Visual area1	Half1	-0.6133
	Visual area2	Half1	0.0854

Post Hoc Tests

Post Hoc Comparisons - Hemisphere

Comparison							
Hemisphere		Hemisphere	Difference	SE	t	df	$p_{bonferroni}$
LH	-	RH	0.0697	0.0109	6.40	198	< .001

Post Hoc Comparisons - Visual area

Comparison							
Visual area		Visual area	Difference	SE	t	df	$p_{bonferroni}$
V1	-	V2	0.02229	0.0147	1.517	318	0.391
V1	-	V3	0.01960	0.0164	1.197	264	0.697
V2	-	V3	-0.00269	0.0131	-0.205	186	1.000

Post Hoc Comparisons - Half

Comparison							
Half		Half	Difference	SE	t	df	$p_{bonferroni}$
dorsal	-	ventral	0.0697	0.0215	3.24	190	0.001

Post Hoc Comparisons - Hemisphere ✻ Visual area

Comparison										
Hemisphere	Visual area		Hemisphere	Visual area	Difference	SE	t	df	$p_{bonferroni}$
LH	V1	-	LH	V2	0.01874	0.0185	1.013	698	1.000
LH	V1	-	LH	V3	0.03349	0.0193	1.737	492	1.000
LH	V1	-	RH	V1	0.07664	0.0170	4.519	872	< .001
LH	V1	-	RH	V2	0.10248	0.0185	5.553	315	< .001
LH	V1	-	RH	V3	0.08234	0.0206	3.992	277	0.001
LH	V2	-	LH	V3	0.01476	0.0173	0.855	511	1.000
LH	V2	-	RH	V2	0.08375	0.0171	4.911	883	< .001
LH	V2	-	RH	V3	0.06360	0.0187	3.403	232	0.012
LH	V3	-	RH	V3	0.04885	0.0166	2.946	827	0.050
RH	V1	-	LH	V2	-0.05790	0.0207	-2.801	380	0.080
RH	V1	-	LH	V3	-0.04315	0.0206	-2.098	304	0.551
RH	V1	-	RH	V2	0.02584	0.0182	1.418	676	1.000
RH	V1	-	RH	V3	0.00570	0.0204	0.280	571	1.000
RH	V2	-	LH	V3	-0.06899	0.0176	-3.921	256	0.002
RH	V2	-	RH	V3	-0.02014	0.0173	-1.162	518	1.000

Post Hoc Comparisons - Hemisphere ✻ Half

Comparison									
Hemisphere	Half		Hemisphere	Half	Difference	SE	t	df	$p_{bonferroni}$
LH	dorsal	-	LH	ventral	0.1509	0.0238	6.33618	280	< .001
LH	dorsal	-	RH	dorsal	0.1509	0.0150	10.08118	611	< .001
LH	dorsal	-	RH	ventral	0.1395	0.0251	5.55089	199	< .001
LH	ventral	-	RH	ventral	-0.0114	0.0139	-0.82181	495	1.000
RH	dorsal	-	LH	ventral	-3.62e−5	0.0231	-0.00156	184	1.000
RH	dorsal	-	RH	ventral	-0.0115	0.0233	-0.49300	257	1.000

Post Hoc Comparisons - Visual area ✻ Half

Comparison									
Visual area	Half		Visual area	Half	Difference	SE	t	df	$p_{bonferroni}$
V1	dorsal	-	V1	ventral	0.09782	0.0253	3.860	354	0.002
V1	dorsal	-	V2	dorsal	0.03567	0.0182	1.956	677	0.763
V1	dorsal	-	V2	ventral	0.10673	0.0233	4.588	231	< .001
V1	dorsal	-	V3	dorsal	0.04838	0.0193	2.502	495	0.190
V1	dorsal	-	V3	ventral	0.08863	0.0275	3.228	210	0.022
V1	ventral	-	V2	ventral	0.00891	0.0186	0.480	704	1.000
V1	ventral	-	V3	ventral	-0.00919	0.0204	-0.451	573	1.000
V2	dorsal	-	V1	ventral	0.06215	0.0302	2.055	276	0.613
V2	dorsal	-	V2	ventral	0.07106	0.0250	2.838	340	0.072
V2	dorsal	-	V3	dorsal	0.01271	0.0171	0.742	499	1.000
V2	dorsal	-	V3	ventral	0.05296	0.0289	1.833	207	1.000
V2	ventral	-	V3	ventral	-0.01810	0.0172	-1.049	510	1.000
V3	dorsal	-	V1	ventral	0.04944	0.0281	1.759	272	1.000
V3	dorsal	-	V2	ventral	0.05835	0.0226	2.581	218	0.158
V3	dorsal	-	V3	ventral	0.04025	0.0249	1.618	332	1.000

Post Hoc Comparisons - Hemisphere ✻ Visual area ✻ Half

Comparison											
Hemisphere	Visual area	Half		Hemisphere	Visual area	Half	Difference	SE	t	df	$p_{bonferroni}$
LH	V1	dorsal	-	LH	V1	ventral	0.18798	0.0303	6.2098	645	< .001
LH	V1	dorsal	-	LH	V2	dorsal	0.02810	0.0240	1.1685	1225	1.000
LH	V1	dorsal	-	LH	V2	ventral	0.19736	0.0284	6.9479	480	< .001
LH	V1	dorsal	-	LH	V3	dorsal	0.07978	0.0245	3.2530	967	0.078
LH	V1	dorsal	-	LH	V3	ventral	0.17519	0.0317	5.5322	362	< .001
LH	V1	dorsal	-	RH	V1	dorsal	0.16680	0.0236	7.0770	1408	< .001
LH	V1	dorsal	-	RH	V1	ventral	0.17446	0.0316	5.5159	469	< .001
LH	V1	dorsal	-	RH	V2	dorsal	0.21005	0.0249	8.4506	834	< .001
LH	V1	dorsal	-	RH	V2	ventral	0.18290	0.0290	6.3033	336	< .001
LH	V1	dorsal	-	RH	V3	dorsal	0.18379	0.0259	7.0889	620	< .001
LH	V1	dorsal	-	RH	V3	ventral	0.16887	0.0327	5.1618	280	< .001
LH	V1	ventral	-	LH	V2	ventral	0.00938	0.0243	0.3855	1239	1.000
LH	V1	ventral	-	LH	V3	ventral	-0.01279	0.0253	-0.5048	1030	1.000
LH	V1	ventral	-	RH	V1	ventral	-0.01352	0.0228	-0.5940	1335	1.000
LH	V1	ventral	-	RH	V2	ventral	-0.00508	0.0243	-0.2090	779	1.000
LH	V1	ventral	-	RH	V3	ventral	-0.01911	0.0265	-0.7203	658	1.000
LH	V2	dorsal	-	LH	V1	ventral	0.15988	0.0349	4.5813	467	< .001
LH	V2	dorsal	-	LH	V2	ventral	0.16926	0.0301	5.6147	637	< .001
LH	V2	dorsal	-	LH	V3	dorsal	0.05168	0.0235	2.2017	1109	1.000
LH	V2	dorsal	-	LH	V3	ventral	0.14710	0.0333	4.4130	356	< .001
LH	V2	dorsal	-	RH	V1	ventral	0.14636	0.0364	4.0203	379	0.005
LH	V2	dorsal	-	RH	V2	dorsal	0.18195	0.0242	7.5074	1464	< .001
LH	V2	dorsal	-	RH	V2	ventral	0.15480	0.0313	4.9492	453	< .001
LH	V2	dorsal	-	RH	V3	dorsal	0.15569	0.0248	6.2685	631	< .001
LH	V2	dorsal	-	RH	V3	ventral	0.14077	0.0344	4.0968	275	0.004
LH	V2	ventral	-	LH	V3	ventral	-0.02216	0.0232	-0.9534	1091	1.000
LH	V2	ventral	-	RH	V2	ventral	-0.01446	0.0228	-0.6348	1337	1.000
LH	V2	ventral	-	RH	V3	ventral	-0.02849	0.0246	-1.1590	614	1.000
LH	V3	dorsal	-	LH	V1	ventral	0.10820	0.0320	3.3864	439	0.051
LH	V3	dorsal	-	LH	V2	ventral	0.11758	0.0276	4.2654	456	0.002
LH	V3	dorsal	-	LH	V3	ventral	0.09542	0.0294	3.2474	590	0.081
LH	V3	dorsal	-	RH	V1	ventral	0.09469	0.0331	2.8614	349	0.295
LH	V3	dorsal	-	RH	V2	ventral	0.10312	0.0283	3.6479	328	0.020
LH	V3	dorsal	-	RH	V3	dorsal	0.10401	0.0229	4.5450	1347	< .001
LH	V3	dorsal	-	RH	V3	ventral	0.08909	0.0304	2.9335	411	0.234
LH	V3	ventral	-	RH	V3	ventral	-0.00632	0.0228	-0.2773	1340	1.000
RH	V1	dorsal	-	LH	V1	ventral	0.02118	0.0293	0.7225	449	1.000
RH	V1	dorsal	-	LH	V2	dorsal	-0.13871	0.0265	-5.2305	842	< .001
RH	V1	dorsal	-	LH	V2	ventral	0.03056	0.0284	1.0754	352	1.000
RH	V1	dorsal	-	LH	V3	dorsal	-0.08703	0.0257	-3.3912	658	0.049
RH	V1	dorsal	-	LH	V3	ventral	0.00839	0.0315	0.2661	300	1.000
RH	V1	dorsal	-	RH	V1	ventral	0.00766	0.0294	0.2603	592	1.000
RH	V1	dorsal	-	RH	V2	dorsal	0.04325	0.0240	1.8055	1221	1.000
RH	V1	dorsal	-	RH	V2	ventral	0.01610	0.0280	0.5753	459	1.000
RH	V1	dorsal	-	RH	V3	dorsal	0.01699	0.0254	0.6697	1026	1.000
RH	V1	dorsal	-	RH	V3	ventral	0.00207	0.0320	0.0646	376	1.000
RH	V1	ventral	-	LH	V2	ventral	0.02290	0.0255	0.8994	761	1.000
RH	V1	ventral	-	LH	V3	ventral	7.33e-4	0.0260	0.0282	676	1.000
RH	V1	ventral	-	RH	V2	ventral	0.00844	0.0242	0.3494	1237	1.000
RH	V1	ventral	-	RH	V3	ventral	-0.00559	0.0260	-0.2148	1082	1.000
RH	V2	dorsal	-	LH	V1	ventral	-0.02207	0.0331	-0.6660	335	1.000
RH	V2	dorsal	-	LH	V2	ventral	-0.01269	0.0293	-0.4335	447	1.000
RH	V2	dorsal	-	LH	V3	dorsal	-0.13027	0.0239	-5.4436	730	< .001

Post Hoc Comparisons - Hemisphere ✻ Visual area ✻ Half

Comparison											
Hemisphere	Visual area	Half		Hemisphere	Visual area	Half	Difference	SE	t	df	$p_{bonferroni}$
RH	V2	dorsal	-	LH	V3	ventral	-0.03485	0.0325	-1.0716	287	1.000
RH	V2	dorsal	-	RH	V1	ventral	-0.03559	0.0335	-1.0615	408	1.000
RH	V2	dorsal	-	RH	V2	ventral	-0.02715	0.0295	-0.9206	596	1.000
RH	V2	dorsal	-	RH	V3	dorsal	-0.02626	0.0233	-1.1264	1094	1.000
RH	V2	dorsal	-	RH	V3	ventral	-0.04118	0.0331	-1.2430	348	1.000
RH	V2	ventral	-	LH	V3	ventral	-0.00771	0.0233	-0.3312	677	1.000
RH	V2	ventral	-	RH	V3	ventral	-0.01403	0.0233	-0.6027	1091	1.000
RH	V3	dorsal	-	LH	V1	ventral	0.00419	0.0322	0.1300	358	1.000
RH	V3	dorsal	-	LH	V2	ventral	0.01357	0.0278	0.4883	329	1.000
RH	V3	dorsal	-	LH	V3	ventral	-0.00860	0.0294	-0.2923	454	1.000
RH	V3	dorsal	-	RH	V1	ventral	-0.00933	0.0324	-0.2882	458	1.000
RH	V3	dorsal	-	RH	V2	ventral	-8.89e−4	0.0275	-0.0323	453	1.000
RH	V3	dorsal	-	RH	V3	ventral	-0.01492	0.0295	-0.5064	594	1.000

References

[1] The jamovi project (2022). *jamovi*. (Version 2.3) [Computer Software]. Retrieved from https://www.jamovi.org.

[2] R Core Team (2021). *R: A Language and environment for statistical computing*. (Version 4.1) [Computer software]. Retrieved from https://cran.r-project.org. (R packages retrieved from MRAN snapshot 2022-01-01).

[3] Gallucci, M. (2019). *GAMLj: General analyses for linear models*. [jamovi module]. Retrieved from https://gamlj.github.io/.
