# Supplementary material for: Variability of visual field maps in human early extrastriate cortex challenges the canonical model of organization of V2 and V3
Source: eLife. 2023 Aug 15;12:e86439. doi: 10.7554/eLife.86439 (PMC10427147; doi:10.7554/eLife.86439)
Supplement: Supplementary file 5. — This file was exported from Jamovi. [file elife-86439-supp5.pdf]

Results - Polar angle

Mixed Model

Model Info

Info	
Estimate	Linear mixed model fit by REML
Call	Mean difference from the average map ~ 1 + Hemisphere + Visual area + Half + Mean difference in curvature + Mean difference in normalized mean BOLD + Mean intra-individual variability + Age_in_Yrs + Hemisphere GM volume + Gender + Hemisphere:Visual area + Hemisphere:Half + Visual area:Half + Hemisphere:Visual area:Half+(1 + Hemisphere + Visual area + Half | HCP_ID)
AIC	13485.256
BIC	13684.334
LogLikel.	-6711.549
R-squared Marginal	0.341
R-squared Conditional	0.627
Converged	yes
Optimizer	bobyqa

Note. (Almost) singular fit. Maybe random coefficients variances are too small or correlations among them too large.

Note. boundary (singular) fit: see help('isSingular')

[3]

Model Results

Fixed Effect Omnibus tests

	F	Num df	Den df	p
Hemisphere	70.9652	1	215	< .001
Visual area	66.2794	2	383	< .001
Half	51.5983	1	207	< .001
Mean difference in curvature	7.9812	1	1807	0.005
Mean difference in normalized mean BOLD	1.0838	1	596	0.298
Mean intra-individual variability	317.7963	1	1361	< .001
Age_in_Yrs	0.9148	1	179	0.340
Hemisphere GM volume	0.0332	1	222	0.856
Gender	0.0432	1	191	0.836
Hemisphere ✻ Visual area	6.4385	2	1461	0.002
Hemisphere ✻ Half	86.6869	1	1518	< .001
Visual area ✻ Half	10.4238	2	1451	< .001
Hemisphere ✻ Visual area ✻ Half	1.1046	2	1453	0.332

Note. Satterthwaite method for degrees of freedom

Fixed Effects Parameter Estimates

Names	Effect	Estimate	SE	95% Confidence Interval Lower	95% Confidence Interval Upper	df	t	p
(Intercept)	(Intercept)	18.5998	0.279	18.0526	19.147	178	66.624	< .001
Hemisphere1	RH - LH	-2.4483	0.291	-3.0178	-1.879	215	-8.425	< .001
Visual area1	V2 - V1	1.9292	0.327	1.2880	2.570	323	5.897	< .001
Visual area2	V3 - V1	3.5370	0.315	2.9188	4.155	288	11.214	< .001
Half1	ventral - dorsal	-2.1113	0.294	-2.6874	-1.535	207	-7.184	< .001
Mean difference in curvature	Mean difference in curvature	0.4025	0.142	0.1233	0.682	1807	2.825	0.005
Mean difference in normalized mean BOLD	Mean difference in normalized mean BOLD	0.1948	0.187	-0.1720	0.562	596	1.041	0.298
Mean intra-individual variability	Mean intra-individual variability	2.9146	0.163	2.5942	3.235	1361	17.827	< .001
Age_in_Yrs	Age_in_Yrs	0.2881	0.301	-0.3023	0.878	179	0.956	0.340
Hemisphere GM volume	Hemisphere GM volume	0.0648	0.355	-0.6318	0.761	222	0.182	0.856
Gender1	M - F	0.1560	0.750	-1.3147	1.627	191	0.208	0.836
Hemisphere1 ✻ Visual area1	RH - LH ✻ V2 - V1	-0.8357	0.479	-1.7737	0.102	1442	-1.746	0.081
Hemisphere1 ✻ Visual area2	RH - LH ✻ V3 - V1	0.9110	0.483	-0.0352	1.857	1462	1.887	0.059
Hemisphere1 ✻ Half1	RH - LH ✻ ventral - dorsal	3.7425	0.402	2.9547	4.530	1518	9.311	< .001
Visual area1 ✻ Half1	V2 - V1 ✻ ventral - dorsal	1.9632	0.481	1.0211	2.905	1455	4.084	< .001
Visual area2 ✻ Half1	V3 - V1 ✻ ventral - dorsal	1.8388	0.481	0.8962	2.781	1456	3.823	< .001
Hemisphere1 ✻ Visual area1 ✻ Half1	RH - LH ✻ V2 - V1 ✻ ventral - dorsal	1.3587	0.958	-0.5195	3.237	1446	1.418	0.156
Hemisphere1 ✻ Visual area2 ✻ Half1	RH - LH ✻ V3 - V1 ✻ ventral - dorsal	0.2971	0.959	-1.5834	2.178	1448	0.310	0.757

Random Components

Groups	Name	SD	Variance	ICC
HCP_ID	(Intercept)	3.36	11.32	0.354
	Hemisphere1	2.61	6.83	
	Visual area1	1.93	3.71	
	Visual area2	1.89	3.58	
	Half1	2.72	7.42	
Residual		4.55	20.66	

Note. Number of Obs: 2172 , groups: HCP_ID 181

Random Parameters correlations

Groups	Param.1	Param.2	Corr.
HCP_ID	(Intercept)	Hemisphere1	0.0827
	(Intercept)	Visual area1	-0.1628
	(Intercept)	Visual area2	0.0907
	(Intercept)	Half1	-0.1172
	Hemisphere1	Visual area1	-0.1400
	Hemisphere1	Visual area2	0.2789
	Hemisphere1	Half1	0.0246
	Visual area1	Visual area2	0.8603
	Visual area1	Half1	-0.2356
	Visual area2	Half1	-0.0177

Post Hoc Tests

Post Hoc Comparisons - Hemisphere

Comparison							
Hemisphere		Hemisphere	Difference	SE	t	df	$p_{bonferroni}$
LH	-	RH	2.45	0.291	8.41	216	< .001

Post Hoc Comparisons - Visual area

Comparison							
Visual area		Visual area	Difference	SE	t	df	$p_{bonferroni}$
V1	-	V2	-1.93	0.328	-5.88	304	< .001
V1	-	V3	-3.54	0.316	-11.18	273	< .001
V2	-	V3	-1.61	0.252	-6.38	183	< .001

Post Hoc Comparisons - Half

Comparison							
Half		Half	Difference	SE	t	df	$p_{bonferroni}$
dorsal	-	ventral	2.11	0.294	7.17	211	< .001

Post Hoc Comparisons - Gender

Comparison							
Gender		Gender	Difference	SE	t	df	$p_{bonferroni}$
F	-	M	-0.156	0.768	-0.203	191	0.839

Post Hoc Comparisons - Hemisphere ✻ Visual area

Comparison									
Hemisphere	Visual area		Hemisphere	Visual area	Difference	SE	t	df	$p_{bonferroni}$
LH	V1	-	LH	V2	-2.347	0.409	-5.74	654	< .001
LH	V1	-	LH	V3	-3.081	0.390	-7.91	589	< .001
LH	V1	-	RH	V1	2.473	0.403	6.14	691	< .001
LH	V1	-	RH	V2	0.962	0.444	2.16	309	0.467
LH	V1	-	RH	V3	-1.519	0.467	-3.25	287	0.019
LH	V2	-	LH	V3	-0.734	0.352	-2.09	617	0.561
LH	V2	-	RH	V2	3.309	0.408	8.12	713	< .001
LH	V2	-	RH	V3	0.828	0.436	1.90	244	0.879
LH	V3	-	RH	V3	1.562	0.399	3.92	672	0.001
RH	V1	-	LH	V2	-4.820	0.482	-10.00	344	< .001
RH	V1	-	LH	V3	-5.555	0.433	-12.84	311	< .001
RH	V1	-	RH	V2	-1.511	0.404	-3.75	634	0.003
RH	V1	-	RH	V3	-3.992	0.406	-9.83	646	< .001
RH	V2	-	LH	V3	-4.043	0.379	-10.67	271	< .001
RH	V2	-	RH	V3	-2.481	0.349	-7.12	600	< .001

Post Hoc Comparisons - Hemisphere ✻ Half

Comparison									
Hemisphere	Half		Hemisphere	Half	Difference	SE	t	df	$p_{bonferroni}$
LH	dorsal	-	LH	ventral	3.983	0.365	10.924	461	< .001
LH	dorsal	-	RH	dorsal	4.319	0.360	11.990	477	< .001
LH	dorsal	-	RH	ventral	4.560	0.425	10.739	229	< .001
LH	ventral	-	RH	ventral	0.577	0.348	1.660	426	0.586
RH	dorsal	-	LH	ventral	-0.337	0.403	-0.836	200	1.000
RH	dorsal	-	RH	ventral	0.240	0.348	0.690	404	1.000

Post Hoc Comparisons - Visual area ✻ Half

Comparison									
Visual area	Half		Visual area	Half	Difference	SE	t	df	$p_{bonferroni}$
V1	dorsal	-	V1	ventral	3.379	0.410	8.234	682	< .001
V1	dorsal	-	V2	dorsal	-0.948	0.405	-2.341	640	0.293
V1	dorsal	-	V2	ventral	0.468	0.420	1.115	262	1.000
V1	dorsal	-	V3	dorsal	-2.618	0.391	-6.699	591	< .001
V1	dorsal	-	V3	ventral	-1.078	0.432	-2.495	252	0.198
V1	ventral	-	V2	ventral	-2.911	0.409	-7.114	655	< .001
V1	ventral	-	V3	ventral	-4.456	0.404	-11.032	639	< .001
V2	dorsal	-	V1	ventral	4.326	0.504	8.587	363	< .001
V2	dorsal	-	V2	ventral	1.415	0.402	3.523	647	0.007
V2	dorsal	-	V3	dorsal	-1.670	0.348	-4.799	596	< .001
V2	dorsal	-	V3	ventral	-0.130	0.427	-0.304	248	1.000
V2	ventral	-	V3	ventral	-1.546	0.347	-4.456	590	< .001
V3	dorsal	-	V1	ventral	5.996	0.476	12.592	342	< .001
V3	dorsal	-	V2	ventral	3.085	0.389	7.923	246	< .001
V3	dorsal	-	V3	ventral	1.540	0.401	3.844	641	0.002

Post Hoc Comparisons - Hemisphere ✻ Visual area ✻ Half

Comparison											
**Hemisphere**	**Visual area**	**Half**		**Hemisphere**	**Visual area**	**Half**	**Difference**	**SE**	**t**	**df**	**$p_{bonferroni}$**
LH	V1	dorsal	-	LH	V1	ventral	4.9739	0.544	9.1508	1235	< .001
LH	V1	dorsal	-	LH	V2	dorsal	-1.7052	0.528	-3.2312	1176	0.084
LH	V1	dorsal	-	LH	V2	ventral	1.9849	0.547	3.6306	656	0.020
LH	V1	dorsal	-	LH	V3	dorsal	-2.2363	0.512	-4.3714	1140	< .001
LH	V1	dorsal	-	LH	V3	ventral	1.0474	0.555	1.8874	608	1.000
LH	V1	dorsal	-	RH	V1	dorsal	4.0686	0.534	7.6241	1256	< .001
LH	V1	dorsal	-	RH	V1	ventral	5.8520	0.588	9.9600	715	< .001
LH	V1	dorsal	-	RH	V2	dorsal	3.8785	0.572	6.7819	735	< .001
LH	V1	dorsal	-	RH	V2	ventral	3.0194	0.577	5.2365	411	< .001
LH	V1	dorsal	-	RH	V3	dorsal	1.0698	0.576	1.8575	609	1.000
LH	V1	dorsal	-	RH	V3	ventral	0.8659	0.607	1.4262	376	1.000
LH	V1	ventral	-	LH	V2	ventral	-2.9890	0.534	-5.5994	1193	< .001
LH	V1	ventral	-	LH	V3	ventral	-3.9265	0.525	-7.4853	1175	< .001
LH	V1	ventral	-	RH	V1	ventral	0.8781	0.523	1.6788	1206	1.000
LH	V1	ventral	-	RH	V2	ventral	-1.9545	0.560	-3.4905	685	0.034
LH	V1	ventral	-	RH	V3	ventral	-4.1080	0.582	-7.0607	623	< .001
LH	V2	dorsal	-	LH	V1	ventral	6.6791	0.626	10.6671	745	< .001
LH	V2	dorsal	-	LH	V2	ventral	3.6901	0.538	6.8580	1214	< .001
LH	V2	dorsal	-	LH	V3	dorsal	-0.5312	0.493	-1.0781	1253	1.000
LH	V2	dorsal	-	LH	V3	ventral	2.7525	0.562	4.8952	644	< .001
LH	V2	dorsal	-	RH	V1	ventral	7.5572	0.673	11.2338	498	< .001
LH	V2	dorsal	-	RH	V2	dorsal	5.5837	0.548	10.1825	1318	< .001
LH	V2	dorsal	-	RH	V2	ventral	4.7245	0.583	8.0981	704	< .001
LH	V2	dorsal	-	RH	V3	dorsal	2.7750	0.556	4.9892	588	< .001
LH	V2	dorsal	-	RH	V3	ventral	2.5710	0.617	4.1687	380	0.003
LH	V2	ventral	-	LH	V3	ventral	-0.9376	0.486	-1.9295	1225	1.000
LH	V2	ventral	-	RH	V2	ventral	1.0344	0.524	1.9756	1209	1.000
LH	V2	ventral	-	RH	V3	ventral	-1.1191	0.550	-2.0342	568	1.000
LH	V3	dorsal	-	LH	V1	ventral	7.2102	0.578	12.4788	671	< .001
LH	V3	dorsal	-	LH	V2	ventral	4.2213	0.516	8.1865	656	< .001
LH	V3	dorsal	-	LH	V3	ventral	3.2837	0.525	6.2554	1166	< .001
LH	V3	dorsal	-	RH	V1	ventral	8.0883	0.607	13.3177	461	< .001
LH	V3	dorsal	-	RH	V2	ventral	5.2557	0.539	9.7591	424	< .001
LH	V3	dorsal	-	RH	V3	dorsal	3.3062	0.524	6.3129	1212	< .001
LH	V3	dorsal	-	RH	V3	ventral	3.1022	0.565	5.4861	647	< .001
LH	V3	ventral	-	RH	V3	ventral	-0.1815	0.523	-0.3473	1205	1.000
RH	V1	dorsal	-	LH	V1	ventral	0.9053	0.562	1.6096	662	1.000
RH	V1	dorsal	-	LH	V2	dorsal	-5.7738	0.597	-9.6777	712	< .001
RH	V1	dorsal	-	LH	V2	ventral	-2.0837	0.587	-3.5519	400	0.028
RH	V1	dorsal	-	LH	V3	dorsal	-6.3050	0.546	-11.5416	698	< .001
RH	V1	dorsal	-	LH	V3	ventral	-3.0213	0.571	-5.2875	399	< .001
RH	V1	dorsal	-	RH	V1	ventral	1.7834	0.524	3.4022	1164	0.046
RH	V1	dorsal	-	RH	V2	dorsal	-0.1901	0.528	-0.3598	1187	1.000
RH	V1	dorsal	-	RH	V2	ventral	-1.0493	0.536	-1.9568	627	1.000
RH	V1	dorsal	-	RH	V3	dorsal	-2.9988	0.527	-5.6887	1176	< .001
RH	V1	dorsal	-	RH	V3	ventral	-3.2028	0.549	-5.8363	595	< .001
RH	V1	ventral	-	LH	V2	ventral	-3.8671	0.583	-6.6282	669	< .001
RH	V1	ventral	-	LH	V3	ventral	-4.8046	0.553	-8.6948	717	< .001
RH	V1	ventral	-	RH	V2	ventral	-2.8326	0.528	-5.3677	1177	< .001
RH	V1	ventral	-	RH	V3	ventral	-4.9861	0.529	-9.4168	1182	< .001
RH	V2	dorsal	-	LH	V1	ventral	1.0954	0.623	1.7581	445	1.000
RH	V2	dorsal	-	LH	V2	ventral	-1.8936	0.561	-3.3755	657	0.051
RH	V2	dorsal	-	LH	V3	dorsal	-6.1149	0.516	-11.8463	773	< .001

Post Hoc Comparisons - Hemisphere ✻ Visual area ✻ Half

Comparison											
Hemisphere	Visual area	Half		Hemisphere	Visual area	Half	Difference	SE	t	df	$p_{bonferroni}$
RH	V2	dorsal	-	LH	V3	ventral	-2.8312	0.561	-5.0507	402	< .001
RH	V2	dorsal	-	RH	V1	ventral	1.9735	0.597	3.3038	657	0.066
RH	V2	dorsal	-	RH	V2	ventral	-0.8592	0.525	-1.6355	1167	1.000
RH	V2	dorsal	-	RH	V3	dorsal	-2.8087	0.491	-5.7247	1249	< .001
RH	V2	dorsal	-	RH	V3	ventral	-3.0126	0.542	-5.5594	581	< .001
RH	V2	ventral	-	LH	V3	ventral	-1.9720	0.506	-3.9003	729	0.007
RH	V2	ventral	-	RH	V3	ventral	-2.1535	0.484	-4.4515	1212	< .001
RH	V3	dorsal	-	LH	V1	ventral	3.9041	0.635	6.1505	442	< .001
RH	V3	dorsal	-	LH	V2	ventral	0.9151	0.575	1.5907	372	1.000
RH	V3	dorsal	-	LH	V3	ventral	-0.0225	0.565	-0.0398	672	1.000
RH	V3	dorsal	-	RH	V1	ventral	4.7822	0.591	8.0918	708	< .001
RH	V3	dorsal	-	RH	V2	ventral	1.9495	0.518	3.7645	664	0.012
RH	V3	dorsal	-	RH	V3	ventral	-0.2039	0.524	-0.3893	1163	1.000

References

[1] The jamovi project (2022). *jamovi*. (Version 2.3) [Computer Software]. Retrieved from https://www.jamovi.org.

[2] R Core Team (2021). *R: A Language and environment for statistical computing*. (Version 4.1) [Computer software]. Retrieved from https://cran.r-project.org. (R packages retrieved from MRAN snapshot 2022-01-01).

[3] Gallucci, M. (2019). *GAMLj: General analyses for linear models*. [jamovi module]. Retrieved from https://gamlj.github.io/.
