# Supplementary material for: Variability of visual field maps in human early extrastriate cortex challenges the canonical model of organization of V2 and V3
Source: eLife. 2023 Aug 15;12:e86439. doi: 10.7554/eLife.86439 (PMC10427147; doi:10.7554/eLife.86439)
Supplement: Supplementary file 6. — The mean deviation in gaze position along the X and Y axes across runs of retinotopic mapping stimuli presentation and individuals is shown for each cluster. We also show the number of individuals with eye-tracking data per cluster, given that eye-tracking data are not available for all individuals. [file elife-86439-supp6.docx]

**Supplementary Table 1 – Gaze position change as a function of cluster assignment.** The mean deviation in gaze position along the X and Y axis across runs of retinotopic mapping stimuli presentation and individuals is shown for each cluster. We also show the number of individuals with eye-tracking data per cluster, given that eye-tracking data is not available for all individuals.

| **Cluster index** | **Mean X average deviation (std)** | **Mean Y average deviation (std)** | **Number of samples with eye-tracking data** |
| --- | --- | --- | --- |
| 1 | 80.00 (59.26) | 101.20 (92.83) | 27 |
| 2 | 43.53 (29.53) | 66.07 (79.10) | 19 |
| 3 | 58.66 (49.40) | 71.99 (73.12) | 22 |
| 4 | 65.50 (98.81) | 70.61 (93.59) | 47 |
| 5 | 63.50 (49.51) | 59.79 (78.15) | 21 |
| 6 | 53.15 (31.89) | 81.16 (92.24) | 11 |
