# Supplementary material for: Variability of visual field maps in human early extrastriate cortex challenges the canonical model of organization of V2 and V3
Source: eLife. 2023 Aug 15;12:e86439. doi: 10.7554/eLife.86439 (PMC10427147; doi:10.7554/eLife.86439)
Supplement: Supplementary file 7. [file elife-86439-supp7.docx]

**Supplementary Table *2*** – Summary of the data used for the analyses described in the main manuscript.

| **File** | **Data type** | **Analysis** |
| --- | --- | --- |
| S1200_7T_Retinotopy181.All.Fit1_PolarAngle_MSMAll.32k_fs_LR.dscalar.nii^[[1]](#footnote-1)^ | Polar angle maps | Individual variability |
| S1200_7T_Retinotopy181.All.Fit1_Eccentricity_MSMAll.32k_fs_LR.dscalar.nii | Eccentricity maps | Individual variability |
| S1200_7T_Retinotopy181.All.curvature_MSMAll.32k_fs_LR.dscalar.nii | Curvature maps | Individual variability / covariate |
| S1200_7T_Retinotopy181.All.Fit1_MeanBOLD_MSMAll.32k_fs_LR.dscalar.nii | Mean BOLD maps | Individual variability / covariate |
| S1200_7T_Retinotopy181.All.**Fit2**_PolarAngle_MSMAll.32k_fs_LR.dscalar.nii | Polar angle maps | Intra-individual variability / covariate |
| S1200_7T_Retinotopy181.All.**Fit3**_PolarAngle_MSMAll.32k_fs_LR.dscalar.nii | Polar angle maps | Intra-individual variability / covariate |
| S1200_7T_Retinotopy181.All.**Fit2**_Eccentricity_MSMAll.32k_fs_LR.dscalar.nii | Eccentricity maps | Intra-individual variability / covariate |
| S1200_7T_Retinotopy181.All.**Fit3**_Eccentricity_MSMAll.32k_fs_LR.dscalar.nii | Eccentricity maps | Intra-individual variability / covariate |

1. “S1200_7T_Retinotopy181.All.(modality)_MSMAll.32k_fs_LR.dscalar.nii” includes collated data from all 181 participants. [↑](#footnote-ref-1)
